# Hydrogen bonding in cytosinium dihydrogen phosphite

**DOI:** 10.1107/S1600536809014020

**Published:** 2009-04-30

**Authors:** Amel Messai, Nourredine Benali-Cherif, Erwann Jeanneau, Dominique Luneau

**Affiliations:** a Laboratoire des Structures, Propriétés et Interactions Inter Atomiques, (LASPI^2^A), Centre Universitaire de Khenchela, 40000 Khenchela, Algeria; b Université Claude Bernard Lyon 1, Laboratoire des Multimatériaux et Interfaces UMR 5615, 69622 Villeurbanne Cedex, France

## Abstract

In the title compound, C_4_H_8_N_3_O_4_P^+^·H_2_PO_3_^−^, the cytosine mol­ecule is monoprotonated and the phospho­ric acid is in the monoionized state. Strong hydrogen bonds, dominated by N—H⋯O inter­actions, are responsible for cohesion between the organic and inorganic layers and maintain the stability of this structure.

## Related literature

For general background, see: Jeffrey & Saenger (1991 ▶); Kabanos *et al.* (1992 ▶); Weber & Craven (1990 ▶); Sivanesan *et al.* (2000 ▶). For hydrogen bonds, see: Blessing (1986 ▶); Masse & Levy (1991 ▶). For related structures, see: Bendheif *et al.* (2003 ▶); Bouchouit *et al.* (2005 ▶); Benali-Cherif, Abouimrane *et al.* (2002 ▶); Benali-Cherif *et al.* (2007 ▶); Benali-Cherif, Benguedouar *et al.* (2002 ▶);  Bendjeddou *et al.* (2003 ▶); Cherouana, Benali-Cherif & Bendjeddou (2003 ▶); Cherouana, Bouchouit *et al.* (2003 ▶); Messai *et al.* (2009 ▶).
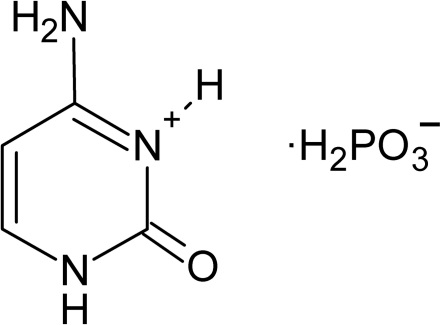


## Experimental

### 

#### Crystal data

C_4_H_6_N_3_O^+^·H_2_PO_3_^−^
*M* *_r_* = 193.10
Triclinic, 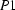

*a* = 4.5625 (3) Å
*b* = 6.4739 (4) Å
*c* = 6.5933 (6) Å
α = 92.934 (4)°
β = 91.236 (4)°
γ = 98.627 (5)°
*V* = 192.21 (2) Å^3^
*Z* = 1
Mo *K*α radiation
μ = 0.34 mm^−1^
*T* = 293 K
0.1 × 0.1 × 0.1 mm

#### Data collection

Nonius KappaCCD diffractometer
Absorption correction: none
1500 measured reflections
1500 independent reflections
1430 reflections with *I* > 2σ(*I*)

#### Refinement

*R*[*F* ^2^ > 2σ(*F* ^2^)] = 0.035
*wR*(*F* ^2^) = 0.084
*S* = 1.13
1500 reflections
112 parameters
4 restraints
H atoms treated by a mixture of independent and constrained refinement
Δρ_max_ = 0.25 e Å^−3^
Δρ_min_ = −0.33 e Å^−3^
Absolute structure: Flack (1983 ▶), 580 with Friedel pairs
Flack parameter: 0.06 (10)

### 

Data collection: *COLLECT* (Nonius, 1997–2000 ▶); cell refinement: *SCALEPACK* (Otwinowski & Minor, 1997 ▶); data reduction: *DENZO* (Otwinowski & Minor, 1997 ▶) and *SCALEPACK*; program(s) used to solve structure: *SIR2004* (Burla *et al.*, 2005 ▶); program(s) used to refine structure: *SHELXL97* (Sheldrick, 2008 ▶); molecular graphics: *ORTEP-3* (Farrugia, 1997 ▶) and *PLATON* (Spek, 2009 ▶); software used to prepare material for publication: *WinGX* (Farrugia, 1999 ▶).

## Supplementary Material

Crystal structure: contains datablocks global, I. DOI: 10.1107/S1600536809014020/gw2061sup1.cif

Structure factors: contains datablocks I. DOI: 10.1107/S1600536809014020/gw2061Isup2.hkl

Additional supplementary materials:  crystallographic information; 3D view; checkCIF report

## Figures and Tables

**Table 1:** Hydrogen-bond geometry (Å, °)

*D*—H⋯*A*	*D*—H	H⋯*A*	*D*⋯*A*	*D*—H⋯*A*
N1—H1⋯O2^i^	0.86	1.86	2.713 (3)	170
O1—H1*A*⋯O3^ii^	0.82	1.75	2.542 (3)	163
N3—H3⋯O3^iii^	0.86	1.94	2.797 (3)	175
N8—H7⋯O2^iii^	0.86	1.89	2.750 (3)	178
N8—H8⋯O1^ii^	0.86	2.29	3.034 (3)	145
N8—H8⋯O3^ii^	0.86	2.44	3.153 (3)	141

Symmetry codes: (i) 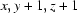; (ii) 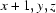; (iii) 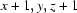.

**Fig. 1.**
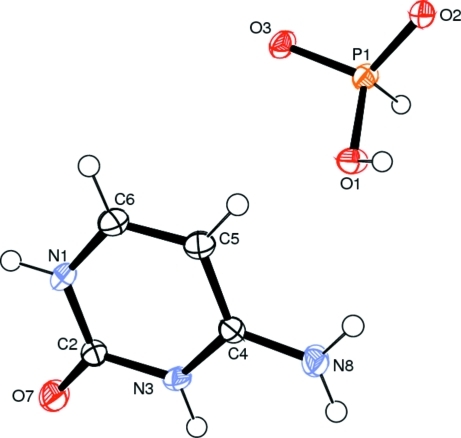
ORTEP view of asymmetric unit.

**Fig. 2.**
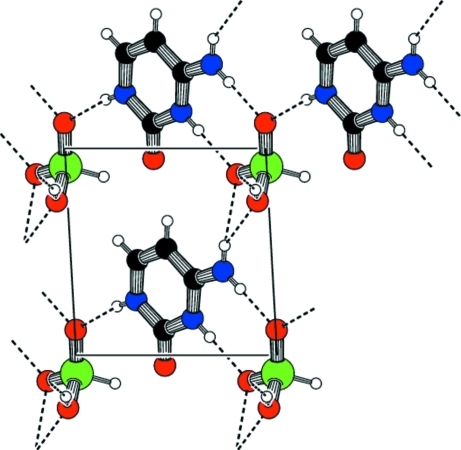
PLUTON view down a axis, showing alternate layers of cations and anions stabilized by N—H···O hydrogen bonds.

## supplementary crystallographic information

### Comment

Studies of metal ion–nucleic acid interactions are of great current interest, since metal ions play a crucial role in the structure and function of nucleic acid and genetic information transfer (Kabanos *et al.*, 1992). Cytosine (6-aminopyrimidin-2-one) is one of the pyrimidines found in deoxyribonucleic acids. It has been the subject of several investigations with the aim of studying the electrostatic properties of its monohydrate form (Weber & Craven, 1990), the relative stabilities of its tautomeric forms and its hydration effects and hydrogen bonding (Sivanesan *et al.*, 2000).

In several crystal structures of purines and pyrimidines with inorganic anions, the structural cohesion is assured by strong hydrogen bonds, as was observed in guaninium sulfate monohydrate (Cherouana, Benali-Cherif & Bendjeddou, 2003) and adeninium perchlorate (Bendjeddou *et al.*, 2003). The potential importance of hydrogen bonding in the structure and function of biomolecules has been well established (Jeffrey & Saenger, 1991); in particular, N—H···O hydrogen bonds are most predominant in determining the formation of secondary structure elements in proteins, base-pairing in nucleic acids and their biomolecular interactions. This structure analysis of cytosinium hydrogenphosphite (I) was undertaken as part of a more general investigation into the nature of hydrogen bonding between organic bases or amino acids and inorganic acids in their crystalline forms (Messai *et al.*, 2009; Benali-Cherif, Abouimrane *et al.*, 2002; Benali-Cherif, Benguedouar *et al.*, 2002; Benali-Cherif *et al.*, 2007).

The asymmetric unit contains one protonated cytosine rings and one hydrogenphosphite anion (Fig. 1). The main feature of the alkyl or aryl ammonium hydrogenphosphite is that the anionic subnetwork is built up through short strong hydrogen bonds (Blessing, 1986) and the organic cations are bonded to the phosphite layers by weaker hydrogen bonds (Masse & Levy, 1991) forming a two-dimensional network of hydrogen bonds (Fig. 2).

The inorganic moiety is a network of H_2_P O_3_^-^ tetrahedra, connected by short and strong hydrogen bonds. Inside these chains each H_2_P O_3_^-^ group is connected to its two adjacent neighbours by strong hydrogen bonds (O5—H2···O3) to build a two-dimensional network along the c direction. Some similarities may be observed between the present atomic arrangement and the corresponding hydrogenphosphites investigated earlier (Bendheif *et al.*, 2003). cytosine is monoprotonated at atom N3. Some base stacking is retained and hydrogen bonding between cytosine rings, as found cytosinium nitrate (Cherouana, Bouchouit *et al.*, 2003), and cytosinium oxalate monohydrate (Bouchouit *et al.*, 2005) are observed. The pyrimidine ring bond distances are, in general, not signicantly different from those found in cytosine or cytosine monohydrate. Each ring is linked to three nitrate anions by strong N—H···O hydrogen bonds *via* atoms N1, N3 and N8. The shortest hydrogen bond is observed between the protonated atom N3 of pyrimidine and atom O3 of hydrogenophosphite.

### Experimental

The title compound (I) was crystallized from a 1:1 aqueous solution of cytosine [4-aminopyrimidine-2(1*H*)-one] and phosphorous acid. Yellow crystals grew after a few days, at room temperature and were manually separated for single-crystal X-ray analysis.

### Refinement

The title compound crystallizes in the non centrosymmetric space group P_1_. All non-H atoms were refined with anisotropic atomic displacement parameters. All H atoms were located in Fourier maps; and treated as riding on their parent atoms, with C—H = 0.93 Å, N—H = 0.86 Å, O—H = 0.82 Å, P—H = 1.30 Å, *U*_iso_ = 1.2Ueq(C,N,P) and *U*_iso_ = 1.5Ueq(O).

### Crystal data

**Table:**

C_4_H_6_N_3_O^+^·H_2_PO_3_^−^	*Z* = 1
*M**_r_* = 193.10	*F*(000) = 100
Triclinic, *P*1	*D*_x_ = 1.668 Mg m^−^^3^
Hall symbol: P 1	Mo *K*α radiation, λ = 0.71073 Å
*a* = 4.5625 (3) Å	Cell parameters from 739 reflections
*b* = 6.4739 (4) Å	θ = 0.7–27.9°
*c* = 6.5933 (6) Å	µ = 0.34 mm^−^^1^
α = 92.934 (4)°	*T* = 293 K
β = 91.236 (4)°	Cubic, yellow
γ = 98.627 (5)°	0.1 × 0.1 × 0.1 mm
*V* = 192.21 (2) Å^3^	

### Data collection

**Table:**

Nonius KappaCCD diffractometer	1430 reflections with *I* > 2σ(*I*)
Radiation source: fine-focus sealed tube	*R*_int_ = 0.0000
horizonally mounted graphite crystal	θ_max_ = 28.0°, θ_min_ = 3.2°
Detector resolution: 9 pixels mm^-1^	*h* = −6→5
CCD rotation images, thick slices scans	*k* = −8→8
1500 measured reflections	*l* = −8→8
1500 independent reflections	

### Refinement

**Table:**

Refinement on *F*^2^	Secondary atom site location: difference Fourier map
Least-squares matrix: full	Hydrogen site location: difference Fourier map
*R*[*F*^2^ > 2σ(*F*^2^)] = 0.035	H atoms treated by a mixture of independent and constrained refinement
*wR*(*F*^2^) = 0.084	*w* = 1/[σ^2^(*F*_o_^2^) + (0.0447*P*)^2^ + 0.0402*P*] where *P* = (*F*_o_^2^ + 2*F*_c_^2^)/3
*S* = 1.13	(Δ/σ)_max_ < 0.001
1500 reflections	Δρ_max_ = 0.25 e Å^−^^3^
112 parameters	Δρ_min_ = −0.33 e Å^−^^3^
4 restraints	Absolute structure: Flack (1983), with how many Friedel pairs?
Primary atom site location: structure-invariant direct methods	Flack parameter: 0.06 (10)

### Special details

**Table:**

Geometry. All e.s.d.'s (except the e.s.d. in the dihedral angle between two l.s. planes) are estimated using the full covariance matrix. The cell e.s.d.'s are taken into account individually in the estimation of e.s.d.'s in distances, angles and torsion angles; correlations between e.s.d.'s in cell parameters are only used when they are defined by crystal symmetry. An approximate (isotropic) treatment of cell e.s.d.'s is used for estimating e.s.d.'s involving l.s. planes.
Refinement. Refinement of *F*^2^ against ALL reflections. The weighted *R*-factor *wR* and goodness of fit *S* are based on *F*^2^, conventional *R*-factors *R* are based on *F*, with *F* set to zero for negative *F*^2^. The threshold expression of *F*^2^ > σ(*F*^2^) is used only for calculating *R*-factors(gt) *etc*. and is not relevant to the choice of reflections for refinement. *R*-factors based on *F*^2^ are statistically about twice as large as those based on *F*, and *R*- factors based on ALL data will be even larger.

### Fractional atomic coordinates and isotropic or equivalent isotropic displacement parameters (Å^2^)

**Table:**

	*x*	*y*	*z*	*U*_iso_*/*U*_eq_	
O7	0.3049 (5)	0.5610 (3)	1.0498 (3)	0.0265 (5)	
N1	0.3442 (5)	0.6856 (3)	0.7331 (4)	0.0213 (5)	
H1	0.2420	0.7833	0.7642	0.026*	
N3	0.5864 (5)	0.4107 (3)	0.8218 (3)	0.0183 (5)	
H3	0.6371	0.3301	0.9118	0.022*	
N8	0.8625 (5)	0.2462 (4)	0.5932 (4)	0.0236 (5)	
H7	0.9227	0.1652	0.6793	0.028*	
H8	0.9180	0.2312	0.4701	0.028*	
C2	0.4021 (5)	0.5545 (4)	0.8791 (4)	0.0177 (5)	
C4	0.6911 (5)	0.3898 (4)	0.6323 (4)	0.0178 (5)	
C5	0.6099 (6)	0.5224 (4)	0.4837 (4)	0.0232 (6)	
H5	0.6711	0.5094	0.3507	0.028*	
C6	0.4412 (6)	0.6684 (4)	0.5406 (4)	0.0228 (6)	
H6	0.3901	0.7593	0.4457	0.027*	
P1	−0.06174 (7)	0.00189 (6)	0.08872 (6)	0.01852 (18)	
O1	0.1928 (4)	0.0549 (3)	0.2547 (3)	0.0209 (4)	
H1A	0.3538	0.0628	0.2004	0.031*	
O2	0.0527 (4)	−0.0044 (3)	−0.1225 (3)	0.0222 (4)	
O3	−0.2857 (4)	0.1487 (3)	0.1256 (3)	0.0214 (4)	
H9	−0.172 (6)	−0.1857 (19)	0.137 (5)	0.026*	

### Atomic displacement parameters (Å^2^)

**Table:**

	*U*^11^	*U*^22^	*U*^33^	*U*^12^	*U*^13^	*U*^23^
O7	0.0323 (11)	0.0300 (11)	0.0203 (11)	0.0138 (8)	0.0056 (9)	0.0032 (8)
N1	0.0274 (12)	0.0176 (11)	0.0212 (13)	0.0108 (9)	−0.0016 (10)	0.0023 (9)
N3	0.0231 (11)	0.0198 (11)	0.0138 (11)	0.0079 (9)	−0.0004 (9)	0.0043 (8)
N8	0.0343 (13)	0.0234 (11)	0.0163 (11)	0.0134 (9)	0.0058 (10)	0.0031 (9)
C2	0.0175 (13)	0.0174 (13)	0.0181 (15)	0.0036 (10)	−0.0010 (10)	−0.0007 (10)
C4	0.0218 (13)	0.0166 (12)	0.0150 (13)	0.0034 (10)	−0.0016 (11)	−0.0005 (10)
C5	0.0327 (14)	0.0224 (13)	0.0165 (13)	0.0094 (11)	0.0002 (12)	0.0042 (10)
C6	0.0308 (15)	0.0188 (13)	0.0193 (15)	0.0056 (11)	−0.0032 (11)	0.0033 (10)
P1	0.0176 (3)	0.0189 (3)	0.0200 (4)	0.0051 (2)	0.0016 (2)	0.0033 (2)
O1	0.0142 (8)	0.0330 (10)	0.0170 (10)	0.0066 (7)	0.0028 (7)	0.0039 (7)
O2	0.0271 (10)	0.0250 (10)	0.0174 (10)	0.0129 (8)	0.0018 (8)	0.0008 (8)
O3	0.0175 (9)	0.0255 (10)	0.0235 (11)	0.0098 (7)	0.0024 (8)	0.0032 (8)

### Geometric parameters (Å, °)

**Table:**

O7—C2	1.220 (3)	C4—C5	1.414 (4)
N1—C6	1.358 (4)	C5—C6	1.349 (4)
N1—C2	1.362 (4)	C5—H5	0.9300
N1—H1	0.8600	C6—H6	0.9300
N3—C4	1.354 (3)	P1—O2	1.498 (2)
N3—C2	1.389 (3)	P1—O3	1.5114 (18)
N3—H3	0.8600	P1—O1	1.5655 (18)
N8—C4	1.321 (3)	P1—H9	1.301 (16)
N8—H7	0.8601	O1—H1A	0.8200
N8—H8	0.8600		
			
C6—N1—C2	122.8 (2)	N3—C4—C5	118.4 (2)
C6—N1—H1	118.6	C6—C5—C4	118.1 (3)
C2—N1—H1	118.6	C6—C5—H5	121.0
C4—N3—C2	123.8 (2)	C4—C5—H5	121.0
C4—N3—H3	118.1	C5—C6—N1	121.5 (2)
C2—N3—H3	118.1	C5—C6—H6	119.2
C4—N8—H7	126.0	N1—C6—H6	119.2
C4—N8—H8	117.2	O2—P1—O3	114.99 (10)
H7—N8—H8	116.8	O2—P1—O1	112.60 (10)
O7—C2—N1	123.7 (2)	O3—P1—O1	108.41 (11)
O7—C2—N3	121.1 (2)	O2—P1—H9	110.0 (14)
N1—C2—N3	115.2 (2)	O3—P1—H9	109.9 (13)
N8—C4—N3	119.0 (2)	O1—P1—H9	99.9 (14)
N8—C4—C5	122.7 (3)	P1—O1—H1A	109.5
			
C6—N1—C2—O7	−176.3 (2)	C2—N3—C4—C5	0.3 (4)
C6—N1—C2—N3	4.5 (3)	N8—C4—C5—C6	−178.0 (3)
C4—N3—C2—O7	177.1 (2)	N3—C4—C5—C6	2.4 (4)
C4—N3—C2—N1	−3.7 (3)	C4—C5—C6—N1	−1.7 (4)
C2—N3—C4—N8	−179.2 (2)	C2—N1—C6—C5	−2.0 (4)

### Hydrogen-bond geometry (Å, °)

**Table:**

*D*—H···*A*	*D*—H	H···*A*	*D*···*A*	*D*—H···*A*
N1—H1···O2^i^	0.86	1.86	2.713 (3)	170
O1—H1A···O3^ii^	0.82	1.75	2.542 (3)	163
N3—H3···O3^iii^	0.86	1.94	2.797 (3)	175
N8—H7···O2^iii^	0.86	1.89	2.750 (3)	178
N8—H8···O1^ii^	0.86	2.29	3.034 (3)	145
N8—H8···O3^ii^	0.86	2.44	3.153 (3)	141

Symmetry codes: (i) *x*, *y*+1, *z*+1; (ii) *x*+1, *y*, *z*; (iii) *x*+1, *y*, *z*+1.
